# Immunization coverage and its determinants among children aged 12 - 23 months in a peri-urban area of Kenya

**DOI:** 10.11604/pamj.2013.14.3.2181

**Published:** 2013-01-02

**Authors:** Lilian Chepkemoi Maina, Simon Karanja, Janeth Kombich

**Affiliations:** 1 Institute of Tropical Medicine and Infectious Diseases, Jomo Kenyatta University of agriculture and Technology. P.O.BOX 62000-00200 Nairobi, Kenya; 2 Kabianga University College, P.O. Box 2030, 20200, Kericho, Kenya

**Keywords:** Immunization, predictors, coverage, Expanded Programme on immunization, determinants, vaccine preventable diseases

## Abstract

**Introduction:**

The institutionalization of strong immunization services over recent years has ensured that today more than 70% of the worlds’ targeted population is reached. In Kenya, approximately 77% of children aged 12-23 months are fully vaccinated with some districts reporting even lower levels of coverage. However, low immunization coverage remains a challenge in low income and high population settings such as Kaptembwo Location, Nakuru district.

**Methods:**

A cross sectional community based survey was undertaken between January and March 2011. Cluster sampling method was employed. Data was collected using pretested interviewer guided structured questionnaires through house to house visits. Data was analyzed in SPSS using descriptive, bivariate and multivariate logistic regression to identify independent predictors of full immunization.

**Results:**

Complete immunization coverage was 76.6%. Coverage for specific antigens was; BCG (99.5%), OPV0 (97.6%), OPV 1(98.7%), OPV2 (96.6%), OPV3 (90.5%), Penta 1(98.9), Penta 2 (96.6%), Penta 3 (90.0%), Measles (77.4%). The drop-out rate between the first and third pentavalent vaccine coverage was 8.9%. Predictors of full immunization included number of children within the family, place of birth of the child, advice on date of next visit for growth monitoring and opinion on the health immunization services offered.

**Conclusion:**

Complete immunization coverage among children aged 12-23 months is still below target. Efforts to improve vaccination coverage must take into account the immunization determinants found in this study. There is need to focus on strengthening of awareness strategies.

## Introduction

There has been a significant rise in the coverage for the six major vaccine-preventable diseases; pertussis, childhood tuberculosis, tetanus, polio, measles and diphtheria since the initiation of Expanded Programme on Immunization in 1974 by the World Health Organization [1].

In developed countries, where accurate recording of immunization and reporting of diseases is in place, most vaccine-preventable diseases are at or near record lows [2]. Worldwide, DTP coverage increased to an estimated 82 per cent by the end of 2008. Polio is on the verge of eradication. Deaths from measles, a major killer disease, declined by 74 per cent worldwide and by 89 per cent in sub-Saharan Africa between 2000 and 2007 [3]. However, absolute numbers of unvaccinated infants are highest in the most populous developing countries, some of which enjoy fairly high rates of immunization coverage [4].

The complete immunization coverage in Kenya in 2003 was 57% and this rose gradually in 2007 to 77% [5]. However, an estimated 35% of newborns had not been immunized in 2006, translating into 0.5 million unvaccinated children within the country. It is also reported that 20% of deaths among children less than five years of age, are caused by measles. In 2006, 1.5 million children were at risk of contracting measles after an upsurge of confirmed measles outbreaks in 39 districts in Kenya [6].

The Kenya Expanded Programme on Immunization recommends that children receive Bacillus Calmette-Guerin (BCG) and Oral Polio Vaccine (OPV) at birth; three doses of Pentavalent vaccine and OPV at 6, 10 and 14 weeks of age; and measles vaccine at 9 months of age. Immunizations are recorded on vaccine cards or booklets obtained from the clinics. Immunization coverage in Nakuru County in 2010 was 66% and a drop-out rate of 10.0% [7]. Reasons for this drop-out rate are still not known. This study therefore sought to find out the coverage rate of children aged 12-23 months in Kaptembwo as well as to identify the factors that influence it so as to propose recommendations for interventions and increase the immunization coverage.

In Kenya, there are few published studies which have been done and with the purpose of establishing factors that are associated with incomplete immunization. This study sought to identify specific factors associated with immunization coverage in order to advance improved intervention, policies/strategies therefore raising overall immunization coverage.

## Methods

### Study site

Nakuru is one of the most populous districts in Kenya and Kaptembwo is the largest low income settlement in this district covering an area of 25sq Km. The population of Kaptembwo is estimated to be 112,937 with approximately 36,404 households [8]. The choice of Kaptembwo as a study site has been influenced by the experience of work conducted by other investigators in similar settings. Emphasis and focus was put on excluded and invisible children [9]. Slum children have been identified amongst the most socially and economically deprived in the community [10] and are therefore in need of health services and especially universal childhood immunization services which are an entry point to provision of other essential health services. While the area is inhabited by people of low social economic status, the provision of health services in the area are far from adequate. Indeed there appears to be no known study conducted in the area on the determinants of immunization status among children aged 12 to 23 months. Therefore this study sought to determine the complete immunization coverage, antigen-specific coverage and factors associated with incomplete immunization.

### Study design

A cross-sectional community-based study was conducted in which 380 mothers/guardians were interviewed and children aged 12-23 months were sampled. The participants had to meet the inclusion criteria of being residents in the study area for a period of not less than 2 years. The study employed a multi-stage study design as the area comprises of 3 Sub-locations namely; Kaptembwo, Githima and Mwariki each having 2 villages. One village from each Sub-location was randomly selected and included in the study. Sample size per village was done proportionate to its population size. Data was collected from mothers or guardians of the selected 12-23 months old children using a pre-tested semi structured questionnaires using a door to door approach. Information collected included the socio-demographic characteristics as well as knowledge, attitude and perceptions of the mothers/guardians towards immunization, immunization status of the children and other factors influencing coverage in the area. Child Health cards were scrutinized to aid in the assessment of the immunization status of the children. In case of absence of the child health cards, mothers/caretakers of the respective children were asked to recall the immunization history.

### Ethical approval

Approval was obtained from Kenya Medical Research Institute (KEMRI) Scientific Steering Committees and KEMRI National Ethical Review Committee. Permission was also sought from the relevant health institution management. Prior consent was obtained in accordance with the ethical guidelines. All stake holders were informed about the study. Informed consent was sought from the study participants before administration of the questionnaire.

### Data management and analysis

The quantitative data from the field was coded and double entered into a computer database designed using MS-Access application and analysis was done using SPSS Statistical Software. Bivariate and Multivariate analysis were conducted to identify independent predictors of immunization coverage. Odds and Adjusted Odds Ratio (OR & AOR) and 95% Confidence Interval (CI) were used to estimate the strength of association between independent variables and the dependent variable. The threshold for statistical significance was set at p<0.05.

The main outcome measures of the study included immunization coverage level and the vaccination levels of the various antigens administered to children. Other measures included the proportion of fully immunized children, proportion of children who had received none or part of the vaccines on the immunization schedule, different levels of knowledge and attitude regarding immunization among the respondents as well as perceived barriers to immunization in the study area.

## Results

### General characteristics of the study participants

Majority of the participants (43.7%) were aged between 20 and 24 years and 29.7% were 25-29 years. Females were more (94.5%) than the males (5.5%). Those who had attained at-least a Primary School education were 58.7%, 33.2% Secondary School Education and 2.6% college or University. Majority of participants 71.2% reported to have between 1 and 3 children while 20% had 4 to six children. Children enrolled in the study who were aged between 13-18 months were 50.3%, 43.4% aged between 19 - 23 months while 59.7% were females and 40.3% were males. Those born at a health facility were 60.5%, 22.4% at home and 17.1% through Traditional Birth Attendants (Table 1).

**Table 1:** Socio-demographic characteristics of the respondents

Variables	N = 380	%
**Age in years**		
<20	31	8.2
20 - 24	166	43.7
25 - 29	113	29.7
30 - 34	48	12.6
35 and above	22	5.8
**Gender**		
Male	21	5.5
Female	359	94.5
**Level of education**		
No education	21	5.5
Primary education	223	58.7
Secondary education	126	33.2
College/ University	10	2.6
**Number of children within the family**		
1 – 3	301	79.2
4 – 6	76	20.0
7 and above	3	0.8
**Occupation**		
Formal employment	26	6.8
Business	99	26
Unemployed	255	67.1

### The immunization coverage in Kaptembwo

The study showed that 291 (76.6%) of the children were fully immunized by card and history. The immunization coverage by card and history was: BCG (99.5%), OPV0 (97.6%), OPV 1(98.7%), OPV2 (96.6%), OPV3 (90.5%), Penta 1(98.9), Penta 2 (96.6%), Penta 3 (90.0%), measles (77.4%). The drop-out rate between the first and third pentavalent vaccine coverage was 8.9%. Access to immunization services in the area is therefore outstanding at 98.9%. The lowest immunization coverage was recorded for Measles (77.4%) while BCG, which is the gateway for EPI was 99.5% (Table 2).

**Table 2:** Immunization coverage among children aged 12-23 months old residing in the study area

Variables	N = 380	%
**Immunization card**		
Present	372	97.9
Absent	8	2.1
**Immunization received**		
BCG	378	99.5
Oral Polio 0	371	97.6
Oral Polio 1	375	98.7
Oral Polio 2	367	96.6
Oral Polio 3	344	90.5
Penta 1 (DTP+Hep B+Hib)	376	98.9
Penta 2(DTP+Hep B+Hib)	367	96.6
Penta 3(DTP+Hep B+Hib)	342	90.0
Measles vaccine	294	77.4

### Socio-demographic characteristics of study participants and immunization coverage

Bivariate Analysis was done to test for the strength of association between categorical variables. All exposure variables (Independent factors) were associated with the dependent variable (Immunization coverage) to determine which ones had significant association. Odds Ratio (OR) and 95% Confidence Interval (CI) were used to estimate the strength of association between independent variables and the dependent variable. The threshold for statistical significance was set at p<0.05. Among the factors that were found to be significantly associated with immunization coverage include; level of education; Primary education (p=0.002) and secondary education (p<0.001), number of children within the family (p<0.001, OR=2.71), Knowledge on immunization (p=0.005), place of birth (p<0.001, OR=2.77), advice on next date of growth monitoring (p=0.001), opinion of health services offered in the area (P=0.011) (Table 3).

**Table 3:** Immunization coverage in relation to socio-demographic characteristics of the respondents

Variables	Fully Immunized (N=291)		Not fully Immunized (N=89)		OR	95% CI		p value
	n	%	n	%		Lower	Upper	
**Age in years**							
<20	24	77.4	7	22.6	0.76	0.19	3.00	0.698
20 - 24	125	75.3	41	24.7	0.68	0.22	2.12	0.503
25 - 29	89	78.8	24	21.2	0.82	0.25	2.66	0.747
30 - 34	35	72.9	13	27.1	0.60	0.17	2.10	0.423
35 and above	18	81.8	4	18.2	Ref.			
**Marital status**							
Married	274	77.6	79	22.4	2.04	0.90	4.63	0.083
Without a spouse	17	63.0	10	37.0	Ref.			
**Level of education**							
No formal education	9	42.9	12	57.1	Ref.			
Primary	171	76.7	52	23.3	4.38	1.75	10.98	0.002
≥Secondary	111	81.6	25	18.4	5.92	2.25	15.57	<0.001
**Number of children within the family**							
1 – 3	243	80.7	58	19.3	2.71	1.59	4.62	<0.001
4 and above	48	60.8	31	39.2	Ref.			
More than 5 Km	29	69.0	13	31.0	Ref.			
**Knowledge score on immunization and vaccination**							
0 - 2	72	77.4	21	22.6	0.52	0.24	1.13	0.100
3 - 5	140	71.4	56	28.6	0.38	0.19	0.75	0.005
6 or more	79	86.8	12	13.2	Ref.			
**Have a reason(s) for which would not take the child for immunization**							
Yes	24	61.5	15	38.5	Ref.			
No	267	78.3	74	21.7	2.27	1.12	4.55	0.019
**Rank of the child within the family**							
1 -2	211	79.3	55	20.7	1.28	0.52	3.16	0.594
3 – 4	59	68.6	27	31.4	0.73	0.28	1.92	0.522
5 and higher	21	75.0	7	25.0	Ref.			
Place of birth Health facility	193	83.9	37	16.1	2.77	1.70	4.50	<0.001
Home/TBAs	98	65.3	52	34.7	Ref.			

### Factors associated with immunization coverage

All independent variables identified to significantly associate with “Immunization coverage” at bivariate analysis were considered together in a Multivariate analysis. This was performed using Binary logistic regression where backward conditional method was specified in order to identify confounders and/or effect modifiers. Adjusted odds Ratio (AOR) with corresponding 95% Confidence Interval (CI) were used to estimate the strength of association between the retained independent predictors of “Full immunization coverage”. Predictors of full immunization coverage in Kaptembwo were identified as; number of children within the family (p=0.001, OR=2.67), Place of birth (p=0.002 OR=2.26), advice on next date for growth monitoring (p=0.003, OR=2.94), Opinion on immunization services offered in the area (p=0.008 OR=2.21) (Table 4).

**Table 4:** Logistic Regression

Predictors	AOR	95% CI		P value
		Lower	Upper	
**Number of children within the family**				
1 – 3	2.67	1.50	4.74	0.001
4 and above	Ref.			
Place of birth of the child				
Health facility	2.26	1.36	3.76	0.002
Home/Traditional Birth Attendant	Ref.			
**Advice on date of next visit for growth monitoring**				
Yes	2.94	1.45	5.95	0.003
No	Ref.			
**Opinion on the health immunization services offered at the area**				
Very good	2.21	1.22	3.98	0.008
Good	Ref.			

**Figure 1:**
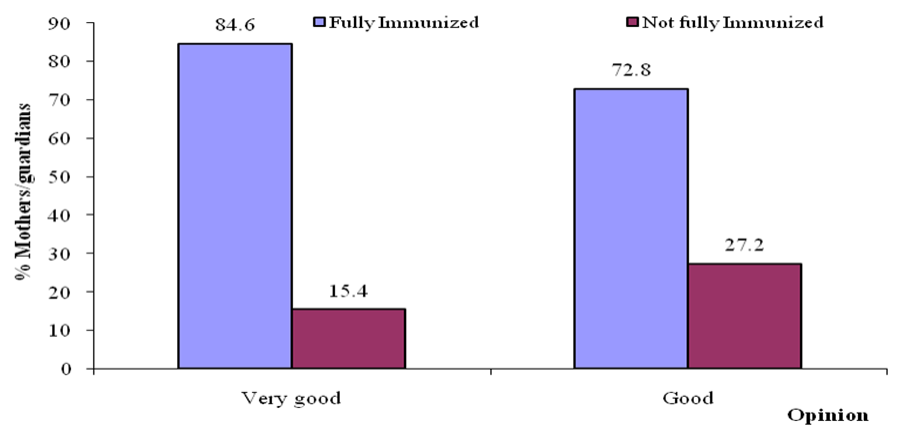
Immunization coverage in relation to opinion on the health immunization services offered within the area by the respondents

On the opinion of respondents on the health immunization services offered in the area, 84.6% of mother/guardians who rated immunization services offered within the area as very good are more likely to have children who have received full immunization compared to those whose mothers/guardians rated immunization services as good (72.8%), (OR=2.05; 95% CI: 1.17 - 3.59; p=0.011) (Figure 1).

## Discussion

This study found that the immunization coverage in Kaptembwo was 67.7% and Penta 1 - Penta 3 drop-out rate of 8.9% which is indicative of good performance. Measles vaccine had the lowest coverage rate of 77.4%. By the age of one year, only 87.5% of children had been fully immunized. The immunization services in the area were rated as very good by 84.6% of the participants and access to these services was supported by the Penta 1 coverage rate of 98.9%. Measles (65.3%) and Polio (69.2%) were two of the most mentioned vaccine preventable diseases by participants.

Maternal education was one of the factors that was significantly associated with immunization coverage. In Kaptembwo, the proportion of fully immunized children of mothers/guardians who had attained secondary school education and above was 81.6% which is higher than those who had attained primary school education (76.7%) and even those with no formal education (42.9%). Previous studies have shown a significant association between immunization coverage and residing in an area with high levels of maternal/guardian education [11].

With over 80% of children delivered at a health facility having received full immunization, the place of birth was found to be one of the predictors of immunization coverage. Though 60.5% of deliveries were conducted at a health facility, the number of home/TBA assisted deliveries accounted for over 30% of all deliveries in the area. This study found that a child who was delivered in a health facility was 2.26 times more likely to receive full immunization compared to one delivered at home (by self) or by a Traditional Birth Attendant. Other investigators have also found similar associations between the place of birth of the child and immunization [12].

Advice given to mothers at health facilities during immunization services was also assessed and participants who recalled having been advised on the next date of growth monitoring had children who were 2.94 times more likely to receive full immunization. Other form of advice that was given at the health facility was reported to be; importance of completing immunization schedules as well as date of next dose of vaccine.

Participants who rated immunization services in the area as being very good (84.6%) were 2.21 times more likely to receive full immunization compared to one whose mother/guardian had a good opinion. Despite the rating of immunization services in the area as being good or very good, reasons given by participants for not taking children for immunization include; unawareness that the child was due for another vaccine, loss of immunization card, child was ill, migration of parents upcountry, service charge and shortage of vaccines at the health facilities. Previous studies have shown an association between full immunization coverage and the said factors [10, 13].

This study was prone to recall bias as the respondents, who did not have the child health cards, were asked to recall the vaccines that were administered to their children. Some of the respondents did not give specific reasons for not taking their children for some of the vaccination. These aspects are therefore considered as limitations to the study.

## Conclusion

The utilization of immunization services in Kaptembwo is good. The participants were aware of the immunization services being offered at the health facilities and importance was identified as being disease preventive. There was, however, no association between mothers/guardians age, marital status and level of education and employment and immunization coverage. There is need to strengthen communication, education and information skills of Health Workers to improve service provision and health education to mothers/guardians. The surveillance and referral systems in the area also need reinforcing so as to identify defaulters of immunization and reduce the drop-out rate.
